# Mental Health Problems in Parents of Children with Congenital Heart Disease

**DOI:** 10.3389/fped.2017.00102

**Published:** 2017-05-08

**Authors:** Gerasimos A. Kolaitis, Maya G. Meentken, Elisabeth M. W. J. Utens

**Affiliations:** ^1^Department of Child Psychiatry, Medical School, “Aghia Sophia” Children’s Hospital, National and Kapodistrian University of Athens, Athens, Greece; ^2^Department of Child and Adolescent Psychiatry/Psychology, Erasmus MC – Sophia Children’s Hospital, Rotterdam, Netherlands; ^3^Research Institute of Child Development and Education, University of Amsterdam, Amsterdam, Netherlands; ^4^Academic Center for Child Psychiatry the Bascule/Department Child and Adolescent Psychiatry, Academic Medical Center, Amsterdam, Netherlands

**Keywords:** children, congenital heart disease, parents, stress, psychopathology

## Abstract

This review will provide a concise description of mental health problems in parents of children with a (non-syndromic) congenital heart disease (CHD) during different stressful periods. Predictors of these problems and also implications for clinical practice will be mentioned. Having a child with CHD can be very stressful for parents, who have to face overwhelming emotions and also extra physical, financial, and other practical challenges. Parental distress has been reported in 30–80% of parents and appears not to be related to severity of CHD. Parental mental health, parenting, the parent–child relationship, and parental quality of life can all be affected. Parents, and especially mothers, are at risk of psychological distress, anxiety, depression, somatization, hopelessness, and posttraumatic stress symptoms, which in turn may influence mother’s responsiveness. In the long term, the majority of parents adapt successfully to living with a child with CHD, but approximately 40% report a need for psychosocial care. These families may be helped by early psychosocial interventions to alleviate stress and reduce children’s emotional and behavioral problems. A holistic approach to early psychosocial interventions should aim at improving coping and enhance parenting. During routine medical checkups, medical professionals should ask about parental stress, family functioning, and psychosocial functioning of the child and, when needed, adequate psychosocial care should be provided.

## Introduction

Approximately 36,000 infants (1% of total live births) are born with a congenital heart disease (CHD) in the USA each year (1). It is well known that due to huge improvements in the medical and surgical treatment of CHD in the last three decades, 85% of infants are expected to survive (2). In a retrospective cohort study, Oster et al. found that 1-year survival for infants with critical CHD improved from 67.4% for the 1979–1993 birth era to 82.5% for the 1994–2005 birth era (*P* < 0.001) (2). Because of the improved survival rates, more and more studies have looked into the psychological outcomes of children with CHD and their parents. Having a child with CHD can be very stressful for parents; the overwhelming emotions and experiences at the time of diagnosis, cardiac surgery, and thereafter may impact parental quality of life and their capacity for optimal parenting. Research indicates that parents, and especially mothers of children with CHD, report mental health problems (such as depression, anxiety, and feelings of guilt), adjustment problems, and poor quality of life more often than parents of healthy children or children with other medical problems (3, 4). These parental mental health problems can be present during different phases of the lives of the children and their medical trajectories. A recent systematic review revealed that up to 30% of parents of children with critical CHD have posttraumatic stress (PTS) symptoms, 25–50% of them reported symptoms of depression and/or anxiety and 30–80% severe psychological distress, particularly shortly after children’s cardiac surgery (5). In addition, parents have to face various extra physical, financial, and other practical challenges.

In this chapter, we give a short overview of parental mental health problems across different stressful or even traumatic periods, e.g., pre- and postnatal diagnosis, the time around cardiac surgery and hospital admissions, and parental well-being on the long term. Due to limited space, this mini review does not have the aim to give a complete systematic review, but rather aims to describe the most prominent problems concerning parents of children with CHD (Table 1).

**Table 1:** **Details of studies included in the mini review**.

Reference	Population studied	Measures	Risk factors	Main findings/types of problems
**Parental mental health problems at the time of diagnosis**				
Lawoko and Soares (3)	*N* = 632 parents of children with congenital heart disease (CHD; 58% women)	Symptom Checklist – 90 – Revised (SCL – 90 – R). The Hopelessness Scale	Gender: mothers had more severe symptoms of depression, anxiety, somatization, and hopelessness than fathers. Parental caregiving burden, feeling dissatisfaction about care, social isolation, and financial difficulties were associated with an elevated risk of long-standing parental psychopathology	Parental depression (18%), anxiety (16–18%), somatization (31–38%), and hopelessness (16%)

Fonseca et al. (4)	*N* = 42 infants with congenital anomalies (40% CHD) and *N* = 42 healthy controls	Symptom Inventory-18, World Health Organization Quality of Life-Brief instrument	Being a mother and postnatal diagnosis are risk factors for more adjustment difficulties	Parents of infants with a congenital anomaly had higher distress than parents of healthy infants

Jackson et al. (6)		Systematic review of 25 studies that were selected, using the PRISMA guidelines	Families with fewer psychosocial resources and lower support are at risk of higher parental psychological distress	Higher anxiety, depression, somatization, and hopelessness in parents of children with CHD compared to parents of healthy children orthose with other diseases

Solberg et al. (7)	*N* = 162 mothers of infants with CHD and *N* = 44,400 mothers of healthy controls within the Norwegian Mother and Child Cohort Study	Hopkins Symptom Checklist (SCL-8)	CHD was a substantial risk factor for parental mental health problems	Mothers of CHD children had increased depression and anxiety compared to controls; mothers of infants with severe CHD had different postpartum mental health compared to healthy controls at 6, 18, and 36 months postpartum

Bevilacqua et al. (8)	*N* = 38 parental couples of infants with CHD	General Health Questionnaire-30 (GHQ-30), Beck Depression Inventory (BDI), Quality of Life: SF-36	Prenatal diagnosis was associated with higher depression in mothers and postnatal diagnosis with more maternal stress	Mothers had higher stress and depression levels, compared to fathers (81.8 versus 60.6 and 45.7 versus 20.0%, respectively)

Cantwell-Bartl and Tibballs (9)	*N* = 16 mothers and *N* = 13 fathers	Structured Clinical Interview for Diagnosis-Clinical Version [posttraumatic stress disorder (PTSD) module]		The majority of parents (88% of mothersand 66% of fathers) had PTSD (only five parents were free of traumatic stress)

Fischer et al. (10)	*N* = 38 neonates	State Trait Anxiety Scale (STAI)	Higher education and less medication associated with higher parental anxiety	Low trait and higher state anxiety scores in parents

Solberg et al. (11)	*N* = 141 mothers of infants with CHD and *N* = 36,437 mothers from the Norwegian Mother and Child Cohort Study	SCL-8	Severity of child’s CHD is associated with higher levels of depression and anxiety symptoms	Mothers of infants with severe CHD are at risk of anxiety and depression from delivery to 36 months postpartum

Landolt et al. (12)	*N* = mothers of 408 children with CHD	Fussy/Difficult Scale from the Infant Characteristics Questionnaire, Child Behavior Checklist, SCL-8	More negative child behavior at 18 months was associated with poorer maternal mental health at 36 months in CHD and controls	CHD explained 31% and 39% of the variance in child and maternal mental health problems

**Parental mental health problems at postsurgery period**				
Woolf-King et al. (5)		A systematic review of 30 studies that were selected using the PRISMA guidelines		30% of parents have PTS symptoms, 25–50% depression/anxiety symptoms, and 30–80% severe distress

Helfricht et al. (13)	*N* = 135 mothers and *N* = 98 fathers of 139 children with CHD undergoing surgery	Posttraumatic Diagnostic Scale	PTS symptom severity at discharge predicted PTSD severity 6 months later	16.4% of mothers and 13.3% of fathers had acute PTSD; 15.7% of mothers and 13.3% of fathers had PTS symptoms

Vrijmoet-Wiersma et al. (14)	*N* = 114 mothers and *N* = 82 fathers of 131 children	Pediatric Inventory for Parents-short form, GHQ, Parental Stress Index-short form, STAI, Child Vulnerability Scale	Number of surgical procedures, time past since last one, and ethnicity were risk factors for higher parental anxiety	Parents of children with CHD had higher levels of perceived vulnerability than parents of healthy children; state anxiety was higher in mothers of children with CHD

Üzger et al. (15)	*N* = parents of 73 patients with CHD undergoing cardiac catheterization	BDI, Beck Anxiety Inventory	Cyanosis: mothers of cyanotic children had more anxiety and depression than mothers of acyanotic children	Increased parental depression and anxiety symptoms in parents of children with CHD

Hearps et al. (16)	*N* = 39 caregivers (28 mothers) of 29 children with CHD	Adapted version of Psychosocial Assessment Tool	Increased risk for psychosocial problems is associated with higher emotional distress (in 38.5% of parents)	61.5% of parents at risk comparable to that of the general population, 35.9% at subclinical level, and 2.6% at clinical risk

Farley et al. (17)	*N* = parents of 52 pediatric heart transplant recipients	Questionnaires on illness-related parenting stress and PTS symptoms		19% of parents had PTSD and almost 40% of them had moderately severe to severe PTS symptoms

Nelson and Gold (19)		A review of descriptive, observational, and controlled studies on pediatric intensive care unit and PTSD	More serious disease was associated with PTSD development. Positive association between children’s PTS symptoms and their parents’ symptoms. Mothers at increased risk to develop PTSD (and more severe PTSD) compared to fathers	PTSD in 5–28% and PTS symptoms in 35–62% of parents of children admitted to intensive care unit

Helfricht et al. (20)	*N* = 61 parents of children following surgery and *N* = 52 patients with an acute cardiac event	German version of Acute Stress Disorder Scale (ASDS)	Surgery versus acute cardiac event	25% of parents of children with CHD met diagnostic criteria for ASD

Franich-Ray et al. (21)	*N* = 77 mothers and *N* = 55 fathers of infants who underwent cardiac surgery before 3 months of age	ASDS	Gender: mothers had higher ASD mean scores than fathers for all symptoms (except dissociation)	33.8% of mothers and 18.2% of fathers had ASD

Van Horn et al. (22)	*N* = 38 mothers of children with CHD aged 3–16 years	Modified Semistructured Interview (developed by Beardslee et al., 1992)	Mothers’ perceptions of medical severity were associated with distress about psychosocial issues postdischarge	Maternal distress, anxiety, and depressed mood decreased postdischarge

López et al (23)	*N* = 40 parents of children with CHD and *N* = 115 parents of healthy children	GHQ, Basic Psychological Needs Scales, Self-Determination Scale, Beck Hopelessness Scale, a socioeconomic survey		Children’s surgery decreased parental hopelessness. Parents of children with CHD had worse GHQ scorings than parents of healthy children

**Longitudinal studies of parental mental health problems**				
Lawoko and Soares (3)	*N* = 632 parents of children with CHD (58% women)	Symptom Checklist – 90 – Revised (SCL – 90 – R). The Hopelessness Scale	Parental caregiving burden, feeling dissatisfaction about care, social isolation, and financial difficulties were associated with a higher risk of long-term parental mental health morbidity	Parental depression (18%), anxiety (16–18%), somatization (31–38%), and hopelessness (16%) at both measurement points

Lawoko and Soares (24)	*N* = 1,092 parents of children with CHD, *N* = 112 parents of children with other diseases, and *N* = 293 parents of healthy children	Symptom Checklist – 90 – Revised (SCL – 90 – R). The Hopelessness Scale	Employment status and financial situation were risk factors for parental distress and hopelessness	Parents of children with CHD at higher risk of distress and hopelessness. Mothers within all parent groups had higher distress and hopelessness than fathers. Fathers of children with CHD were doing worse than fathers of the other groups

Berant et al. (25)	*N* = 63 mothers of newborns with CHD	Mothers’ interview on mental health and attachment style, Children’s Apperception Test	Maternal avoidant attachment at initial assessment was the best predictor of worsening of her mental health at 7-year follow-up	Mothers of children with severe CHD were more vulnerable in terms of their mental health

Menahem et al. (26)	*N* = parents of 39 children	Parents were assessed (e.g., mental health, locus of control) prior to and 12–50 months following their children’s surgery		Mothers with increased anxiety and a tendency to attribute events to chance greater than normal; their anxiety decreased at follow-up

## Parental Mental Health Problems Around the Time of Child’s CHD Diagnosis

Several studies showed that during the period of diagnosis, parents of children with CHD experience more psychopathology (e.g., anxiety, depression, and somatization) compared to parents of children with other medical illnesses or healthy controls (3, 6, 7). Parents can experience difficulties at different time points. One possible stressful period is the time around the child’s diagnosis.

As to timing of diagnosis of CHD (e.g., prenatal, postnatal), Fonseca et al. showed that parents of children with a congenital anomaly (40% of which were CHD) were more distressed compared to parents of healthy children, even if they had similar quality of life (4). Interestingly, learning the diagnosis in the prenatal period was related to a higher maternal quality of life compared to receiving the diagnosis after the baby was born.

Bevilacqua et al. found no differences in stress and depression levels in both fathers and mothers, who received the diagnosis of CHD in their child prenatally or postnatally (8). However, mothers who had received the diagnosis prenatally were more depressed, while those who had received a postnatal diagnosis were more stressed. In this study, parental self-reported stress and depression levels within 2 weeks after hospitalization of their infants in the first 3 months of life were significantly higher in mothers compared to fathers.

In a study of Cantwell-Bartl and Tibballs, of the total 18 parents whose infants were diagnosed with hypoplastic left heart syndrome (HLHS) *in utero*, eight of nine mothers and six of nine fathers had posttraumatic stress disorder (PTSD) (9). Of those parents whose infants were diagnosed with HLHS postbirth, six of seven mothers had acute stress disorder (ASD) and one mother had PTSD. Furthermore, two of the four fathers had ASD and one father had PTSD. These parents were clinically assessed with a semistructured interview and the PTSD module of the Structured Clinical Interview for Diagnosis. Only five parents were free of traumatic symptoms. This was the first study with parents of infants with HLHS in the ICU. The high prevalence of traumatic stress of parents in this study is related to the multiple stressors experienced by them, including the CHD diagnosis received after birth of their infant (for 50% of parents) and the life-threatening nature of HLHS, the ICU environment, and surgery.

Fischer et al. studied parental anxiety levels during the first month of their neonates’ life with CHD (upon hospital discharge), using the State Trait Anxiety Scale (STAI) (10). They found low (5% with significant and 2% with borderline) trait anxiety scores, indicating stable personality levels of anxiety in caregivers, whereas higher numbers of caregivers reported clinically significant (5%) and borderline (14%) state anxiety. Higher education was associated with higher level of state and trait anxiety.

In a Norwegian Mother and Child Cohort Study (*n* = 36,437), Solberg et al. studied a subgroup of 141 children with CHD. They found that postpartum mental health of mothers of infants with severe (but not mild/moderate) CHD was different compared to that of cohort controls at 6, 18, and 36 months postpartum. The mothers of CHD children had been experiencing significantly elevated levels of depression and anxiety symptoms (7, 11). In the same cohort, CHD was a substantial risk factor for parental mental health problems in children and their mothers at all time points (12). Both familial and individual factors contributed to risk for developing mental health problems, and mutual influences between mother’s and child’s mental health at 18 and 36 months over time were found.

In sum, despite different methodologies, most studies agree that the period of the child’s CHD diagnosis is generally a stressful period for parents, which may jeopardize the parental mental health. Nevertheless, the mentioned studies have limitations such as small sample size (8–10), reliance on retrospective memory, low participation rate and attrition (7, 11), oversimplification in CHD severity grading (7, 11), use of self-reports, lack of clinical assessment of parental mental health problems (8, 11), and lack of data on possible confounding factors (11).

## Parental Mental Health Problems after Child’s Cardiac Surgery

Parents of children with CHD undergoing cardiac surgery may also be at increased risk for psychological malfunctioning particularly in the weeks and months immediately following cardiac surgery (5, 13). In the study of Vrijmoet-Wiersma et al., predictive factors for increased parental anxiety appeared to be: the time interval since last procedure, the number of surgeries, and ethnicity (14).

Preprocedural mental health of parents of patients with (a)cyanotic CHD was studied by Üzger et al. (15). They found that an upcoming angiography was associated with depression and anxiety in parents of children with CHD. Mothers of children with cyanotic CHD had significantly higher levels of depression and anxiety compared to mothers of children with acyanotic CHD.

In Hearps et al.’s sample, the majority of parents appeared to have adjusted to the acute stress of their infant’s CHD 4 weeks following cardiac surgery. However, 38.5% of them were classified at increased psychosocial risk [35.9% at a targeted (/subclinical) and 2.6% at a clinical level]. This risk was measured using the Psychosocial Assessment Tool (PAT), a brief parent report screener that was adapted to include also sleeping, feeding, crying, and bonding difficulties. PAT scores were associated with higher levels of emotional distress compared to universal psychosocial risk (the lowest 61.5% of parents) (16). As the authors report, the distribution of risk for psychosocial problems in parents of CHD children undergoing surgery is comparable to that of parents of children with other serious pediatric diagnoses such as pediatric cancer. There were no differences between families of infants who received prenatal versus postnatal diagnosis or single ventricle versus biventricular repair. In addition, a higher parent education significantly predicted a lower total psychosocial risk score.

Farley et al. found a PTSD prevalence of 19% in parents of children who underwent pediatric heart transplantation (17). This is a clearly heightened risk in comparison to a PTSD lifetime prevalence of 5.6% in the general population (18). This high rate of PTSD is comparable to that of parents, following their child’s admission to the pediatric intensive care unit (10.5–21%) (19). Fifty-six percentage of the CHD parent sample showed moderate levels of PTSD symptoms and 39% indicated moderately severe to severe PTSD symptoms.

Helfricht et al. reported that acute PTS symptoms in parents following discharge from hospital after cardiopulmonary bypass surgery in their child are a major risk factor for the development of chronic PTSD. Their research showed that following discharge, 16.4% of mothers and 13.3% of fathers of CHD children met diagnostic criteria for acute PTSD, using the Posttraumatic Diagnostic Scale. Another 15.7% of mothers and 13.3% of fathers experienced significant PTS symptoms. Six months after surgery, PTSD rates were 14.9 and 9.5%, respectively. In another study, Helfricht et al. found that 25% of parents of children with CHD met diagnostic criteria for ASD assessed with the German Acute Stress Disorder Scale (20).

Almost similar levels of ASD were found by Franich-Ray et al. in 77 mothers and 55 fathers of infants (younger than 3 months old), 1 month after their child was discharged from hospital following cardiac surgery (21). More specifically, one-third of mothers and almost one-fifth of fathers experienced ASD symptoms. Most of them experienced at least one symptom at a clinical level, while dissociative symptoms were the most commonly experienced group of symptoms.

Van Horn et al. studied mothers of children with CHD and their concerns during hospitalization and 2–4 weeks after discharge from hospital (22). Distress due to concerns decreased postdischarge, as did mother’s anxiety and depressed mood.

In a Latin American study (Chile), parents of children with CHD had a decreased well-being (measured with the General Health Questionnaire-12) compared to parents of healthy children. On the other hand, they had a similar level of agency (a concept from developmental studies defined as “the ability to act on behalf of what you value and have a reason to value”) (23). Their children’s surgery significantly decreased parental feelings of hopelessness, but had no influence on their well-being or agency.

In sum, most of the reviewed studies show that in the period surrounding a child’s cardiac surgery, parents are at elevated risk for developing mainly traumatic reactions, i.e., ASD and PTSD, but also anxiety and depression symptoms; psychological distress may gradually decrease following cardiac surgery. Limitations of reviewed studies include, e.g., small sample size or use of non-standardized instruments (23), underestimation of ASD (21), assessment of mental health symptoms “only,” and not of specific psychiatric diagnoses (13–15, 17, 20, 21, 23).

## Long-Term Parental Mental Health Problems

Several studies investigated parental mental health problems at longer term (after at least 1 year or longer thereafter) following diagnosis or cardiac surgery of their child. In a longitudinal study, Lawoko and Soares studied psychological morbidity and its determinants in parents of children with CHD, with a 1-year follow-up interval. Parents reported a variety of psychological problems: depression (18%), anxiety (16–18%), somatization (31–38%), and hopelessness (16%) during both measurement points. Moreover, 7–22% reported persisting problems during the 1-year follow-up period. Mothers reported more severe mental health problems than fathers. Children’s clinical severity did not explain parents’ psychological morbidity over time. Nevertheless, parental caregiving burden, feeling dissatisfaction about care, social isolation, and financial difficulties were associated with an elevated risk of long-standing parental psychopathology. In their previous study, the same researchers found that parents of children with CHD overall were at higher risk of distress and hopelessness than parents of children with other diseases and parents of healthy children (24). Across all parent groups, mothers had higher levels of distress and hopelessness than fathers, with the highest levels among mothers of children with CHD compared to mothers in the other groups. Fathers of children with CHD were doing worse than fathers belonging to the other groups.

In a 1-year and 7-year follow-up study of children with CHD, maternal avoidant attachment at the time of diagnosis was the best predictor of worsening of mothers’ mental health and maternal satisfaction over this period, especially in a subgroup of whose children had severe CHD (25). In addition, mothers’ attachment insecurities to their own and their children’s psychological functioning (both anxiety and avoidance) at the time of diagnosis were associated with their children’s emotional problems and children’s poor self-image 7 years later.

In the study of Menahem et al., a substantial increase in the emotional distress, e.g., anxiety of mothers of children with CHD at the time of surgery significantly resolved by 12–50 months following the surgery while they still seemed not to feel in “control” at follow-up (26). At baseline, these mothers reported increased anxiety and a tendency to attribute events to luck and/or chance greater than community norms.

In sum, the few longitudinal studies on mental health problems of parents with CHD available show conflicting results, i.e., decline of parental symptoms over time or persistence, especially in more severe CHD cases.

## Clinical Implications—The Need for Psychosocial Care

Despite high variability in methodologies and measurements used in outcome studies, it can be concluded that parents of children with CHD experience numerous stresses and mental health problems. High percentages of them show traumatic stress, anxiety, depression, and other psychiatric morbidities (5, 9, 22). Levert et al. have recently reported that more than 40% of parents and more than 50% of their children with CHD reported a need for psychosocial care on each of five domains studied, i.e., physical/medical, emotional, social and educational/occupational functioning, and health behavior (27). Needs for psychosocial care for parents themselves were highest for parents of 0–12-year-old children. Parents and/or patients reported that they would like to be referred to mental health professionals in case of problems on the domains studied.

The PICU environment, where also the diagnosis is given for many children with CHD, may impact the parent–infant attachment and parental adaptation. The PICU staff may, therefore, help parents in dealing with their new traumatic situation (9). This can be done by providing parents information and psychoeducation, involving them in taking care of their infant as much as possible and strengthening their role as parents, to enhance bonding with their child. Also, other studies point to the need for providing support both to children/adolescents with CHD and their parents, especially mothers (12). There is a need for early identification and screening of parents at risk of stress and mental health problems. Specific interventions to improve parental coping and adjustment are needed. Practitioners working with these children and families should ask about, e.g., parental mental health, stress, and family functioning, in the context of routine medical checkups (28). In this modern digital era, we recommend to screen for mental health problems and parental stress during outpatient consultations, by having parents complete questionnaire digitally on an iPad, in the waiting room during outpatient consultations.

Considering the findings of studies on psychosocial interventions to promote adjustment in families of child with CHD, a holistic approach is recommended (6). The pediatric cardiology group from Belfast (United Kingdom) has highlighted the importance of maternal mental health for child behavioral outcomes at 1-year follow-up. Their psychosocial intervention has been shown to have a positive impact on maternal mental health and functioning of families with children with CHD (29).

Finally, parents and families can be helped by educational interventions such as the use of narrative therapy, strengthening protective factors, cognitive behavioral techniques (relaxation, helpful thoughts, and cognitive restructuring), and provision of psychoeducation to deepen parents’ understanding of their child with CHD (3).

## Conclusion

Despite great methodological variability between reviewed studies, the majority of studies show that parents, and especially mothers, of children with CHD are at higher risk and experience a variety of mental health problems (e.g., PTS, anxiety, depression) at different time periods of their offspring medical condition. Those parents with mental health problems can be helped by mental health professionals. In addition, prospective studies of parental mental health problems, with larger samples of families and use of standardized instruments and interviews.

## Author Contributions

All the authors (GK, MM, and EU) have substantially contributed to the conception of the work. GK has drafted the manuscript, and MM and EU revised it. All the authors (GK, MM, and EU) have made a final approval of the version to be published and have agreed to be accountable for all aspects of the work in ensuring that questions related to the accuracy or integrity of any part of the work are appropriately investigated and resolved.

## Conflict of Interest Statement

The authors declare that the research was conducted in the absence of any commercial or financial relationships that could be construed as a potential conflict of interest.
